# The Role of Lasers in Enhancing Growth Factor-Mediated Regenerative Endodontic Procedures: A Systematic Review

**DOI:** 10.7759/cureus.105801

**Published:** 2026-03-24

**Authors:** Andria Dsouza, Kiran Kumar N., Savitha B Naik, Vaishnavi Kashim, Ansha Jasmin S.

**Affiliations:** 1 Conservative Dentistry and Endodontics, Government Dental College and Research Institute Bangalore, Bangalore, IND

**Keywords:** dental pulp regeneration, growth factors, lasers, photobiomodulation therapy, regenerative endodontics, stem cells

## Abstract

Regenerative endodontics focuses on biologically restoring the pulp-dentin complex in immature permanent teeth affected by necrosis. Endogenous growth factors (GFs) such as vascular endothelial growth factor (VEGF), transforming growth factor-β (TGF-β), fibroblast growth factor (FGF), platelet-derived growth factor (PDGF), and bone morphogenetic proteins (BMPs) play a key role in regulating cellular processes involved in tissue repair and regeneration. Recently, laser technologies have emerged as promising adjuncts for enhancing regenerative outcomes by stimulating cellular activity and GF release. This systematic review aimed to evaluate the influence of laser applications on GF expression and related regenerative outcomes in human dental tissues. The review was conducted following Preferred Reporting Items for Systematic Reviews and Meta-Analyses (PRISMA) guidelines and included human-based in vitro, ex vivo, and in vivo studies that investigated the effects of laser energy on GF activity during regenerative endodontic procedures (REPs). Risk of bias was assessed using the Quality Assessment Tool for In Vitro Studies (QUIN). 15 studies fulfilled the inclusion criteria. Diode lasers with wavelengths ranging from 450-980 nm were the most commonly used, while two studies evaluated Er:YAG lasers. Most studies demonstrated increased expression of GFs such as VEGF, TGF-β1, PDGF, and BMPs, along with enhanced markers of odontogenic, osteogenic, and neurogenic differentiation. However, heterogeneity in laser parameters and experimental designs limited direct comparison across studies. Within the limitations of predominantly preclinical evidence, laser therapy appears to enhance biological signaling pathways associated with pulp-dentin regeneration. Nevertheless, well-designed clinical trials are required to establish standardized protocols and confirm long-term clinical outcomes in regenerative endodontics.

## Introduction and background

Regenerative endodontics has emerged as a promising frontier in dental science, offering the possibility of biologically restoring the pulp-dentin complex in immature permanent teeth affected by necrosis [1]. As an alternative to traditional methods that focus on mechanical debridement and obturation, regenerative endodontic procedures (REPs) aim to harness the body’s healing mechanisms, particularly the activity of endogenous growth factors (GFs), to promote tissue repair, continued root development, and restoration of vitality [2].

Immature permanent teeth with necrotic pulps pose a complex endodontic challenge. Unlike mature permanent teeth, immature permanent teeth have open apical foramina and thin, fragile dentinal walls, and as such, conventional root canal treatment is not indicated for immature permanent teeth. Indeed, the objectives of REPs in immature permanent teeth are not only confined to disinfection of the root canal system but also include creating a biological environment conducive for root maturation, apical closure, and, if possible, pulp sensibility [1,2]. This is not an easy task, as it requires proper regulation of stem cells, scaffolds, and GFs, which cannot be effectively controlled using conventional endodontic treatment alone.

GFs, including vascular endothelial growth factor (VEGF), transforming growth factor-β (TGF-β), fibroblast growth factor (FGF), platelet-derived growth factor (PDGF), and bone morphogenetic proteins (BMPs), play a pivotal role in REP. These signaling molecules are essential for processes such as angiogenesis, stem cell recruitment, and cellular proliferation. The release and activation of these factors ultimately determine the success of REP [2-4].

In conventional regenerative endodontic techniques, one of the major challenges lies in the controlled release of these GFs. Traditional methods often fail to provide precise regulation of the GFs' release, resulting in suboptimal healing or inconsistent tissue regeneration. This can be due to factors such as limited access to the targeted tissue, inefficient delivery mechanisms, and the inability to maintain an appropriate concentration of GFs over time. Additionally, the inflammatory response triggered by conventional procedures can complicate the natural healing process, potentially hindering the desired outcomes [5].

In recent years, laser technologies have emerged as a promising adjunct to conventional REP methods. Lasers, such as Er:YAG, Nd:YAG, and diode lasers, have demonstrated the ability to stimulate GF expression, modulate local inflammation, and enhance cellular responses [5]. One of the most significant advancements is the use of low-level laser therapy (LLLT), which not only accelerates the biological processes necessary for tissue regeneration but also offers a more controlled and targeted approach for GF activation [6-10].

By transitioning from conventional methods to laser-assisted REP, clinicians can achieve more efficient and predictable results. The precision of laser application allows for a more favorable microenvironment that enhances the release of GFs like VEGF, TGF-β, BMPs, and PDGF, promoting better angiogenesis, stem cell recruitment, and cellular proliferation. Furthermore, lasers can improve the overall biological conditions necessary for tissue regeneration [5-10].

Although several experimental studies suggest that laser application can influence stem cell behavior and stimulate the release of GFs, the available evidence remains limited and heterogeneous. Currently, no standardized framework clearly explains how laser-assisted procedures influence the biological outcomes of regenerative endodontics. In particular, the role of laser energy in activating key signaling molecules such as BMPs, VEGF, TGF-β, and PDGF and their interactions with dental stem cells requires further clarification. These GFs are essential regulators of cellular migration, proliferation, and differentiation during pulp-dentin regeneration [11].

Acknowledging the biological significance of these signaling pathways, it is necessary to assess the contribution of laser technology in enhancing regenerative outcomes through GF modulation. This systematic review was conducted to consolidate existing evidence from human-focused studies, including in vitro experiments using human dental tissues and clinical investigations involving regenerative endodontic protocols. Its primary aim is to elucidate the role of laser application in modulating GF expression and associated regenerative mechanisms, thereby contributing to a clearer understanding that can inform clinical practice and support biologically based advancements in endodontic therapy.

## Review

Methodology

Protocol and Reporting Guidelines

The Preferred Reporting Items for Systematic Reviews and Meta-Analyses (PRISMA) standards were followed in the design and execution of this systematic review [12]. Under the ID CRD420251078541, the review methodology was prospectively filed with the International Prospective Register of Systematic Reviews (PROSPERO). Prior to guaranteeing rigor and openness, all methodological choices, including search, selection, and synthesis tactics, were established.

Eligibility Criteria

This review considered studies that explored laser applications in enhancing GF activity and regenerative responses in human samples. Studies were eligible if they evaluated the role of laser application in conjunction with biologically based regenerative protocols. Both in vivo (clinical) and in vitro studies using human-derived dental stem cells were included, provided they measured outcomes related to GF activity or regeneration.

Interventions of interest involved the use of lasers, including Er:YAG, Nd:YAG, diode, CO₂, or LLLT. These lasers must have been applied as part of the regenerative protocol, specifically for disinfection, dentin conditioning, stimulation of GF release, or enhancement of stem cell activity.

The primary outcomes of interest included changes in GF expression (e.g., VEGF, TGF-β, PDGF, FGF). The secondary outcomes focused on clinical and radiographic regenerative indicators such as root lengthening, apical closure, and pulp vitality.

Studies focusing exclusively on soft tissue applications of lasers, those lacking reported outcomes related to GF activity, or those with insufficiently described laser protocols were excluded. In addition, case reports, narrative reviews, expert opinions, conference abstracts, and studies published in languages other than English were also excluded from this review.

Search Strategy

The databases PubMed, MEDLINE, Embase, Scopus, Cochrane Central Register of Controlled Trials (CENTRAL), and Science Citation Index were used in a thorough electronic search. Key terms including "Regenerative Endodontics," "Laser Therapy," "Growth Factors," "Photobiomodulation," "Stem Cells," and "Immature Permanent Teeth" were combined using the search strategy's Boolean operators (AND, OR, NOT). Only English-language publications were taken into consideration, but there were no date restrictions. The included articles' reference lists were manually examined to find any more relevant research.

Study Selection Process

All retrieved records were independently screened by two reviewers. Initial screening was performed at the title and abstract level, followed by full-text evaluation of potentially relevant articles. Discrepancies during the selection process were resolved through discussion, and a third reviewer was consulted if consensus could not be reached.

Data Extraction

Data from the included studies were extracted into a standardized Excel spreadsheet specifically designed for this review. Key fields included: Serial Number, Study ID/Author (Year), Study Design, Cell/Tissue Type, Laser Type and Wavelength, Energy Parameters, GFs/Markers Measured, Key Outcomes, Regenerative Indicators, Study Conclusion. The extraction was performed by one reviewer and independently cross-verified by a second reviewer to ensure accuracy and completeness.

Risk of Bias Assessment

The risk of bias of the included in vitro studies was evaluated using the Quality Assessment Tool for In Vitro Studies (QUIN) checklist. The QUIN assesses methodological rigor across multiple domains including study design, sample characterization, control groups, blinding, statistical analysis, and reproducibility. Each criterion is scored to generate an overall risk-of-bias rating [13]. Two reviewers independently performed the assessment, and disagreements were resolved through discussion.

Data Synthesis

Data synthesis was primarily descriptive, structured around the predefined outcomes and themes. All included studies were summarized in tabular and narrative formats using the Excel extraction sheet. Due to the heterogeneity in study designs, laser parameters, GF types, and outcome measures, a formal meta-analysis was not performed. Instead, findings were qualitatively synthesized to identify consistent patterns, potential benefits, and knowledge gaps in the use of lasers for enhancing GF-mediated regenerative outcomes.

Results

Study Selection

**Figure 1:**
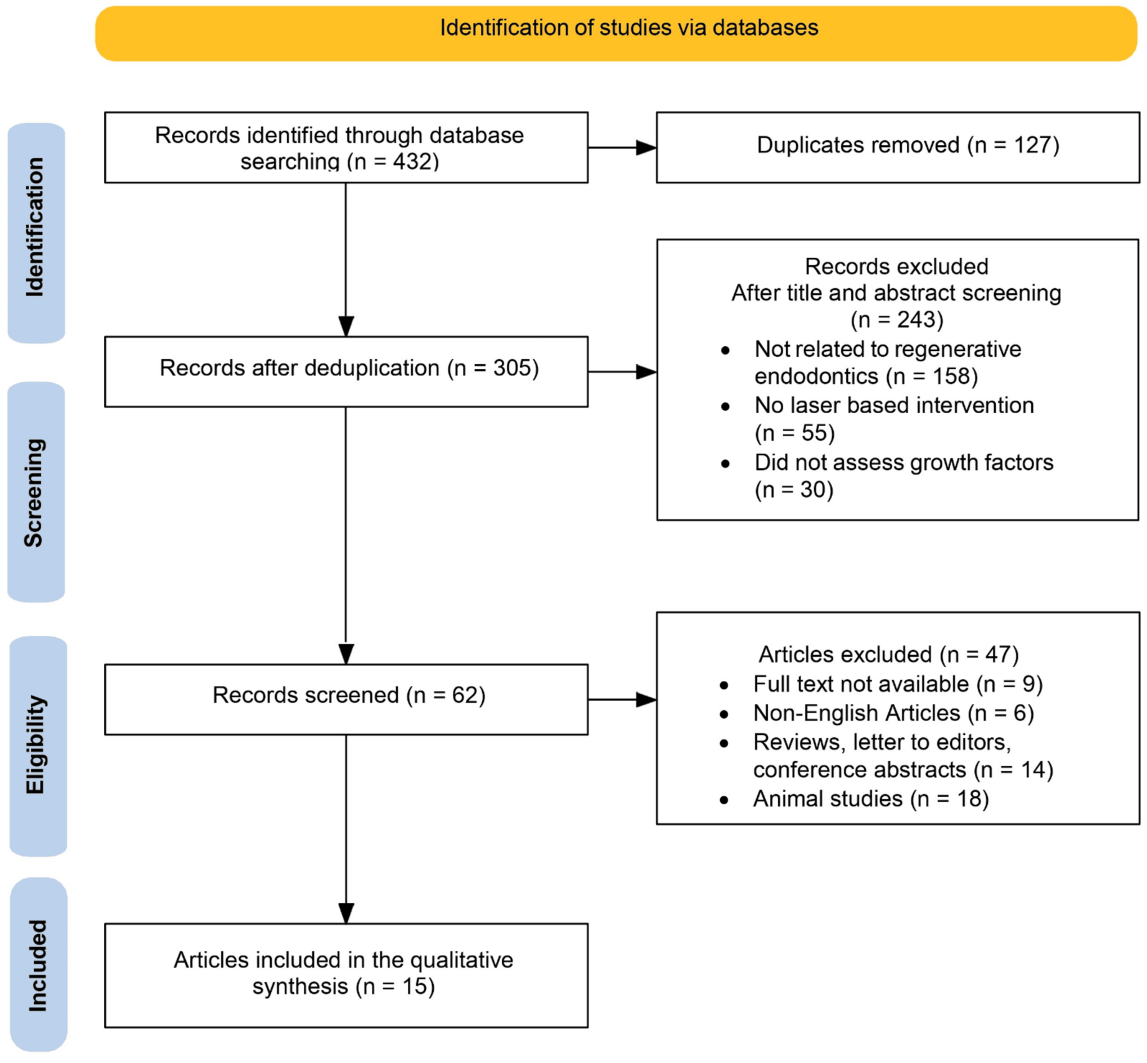
PRISMA flowchart summarizing the study selection process PRISMA: Preferred Reporting Items for Systematic Reviews and Meta-Analyses

A total of 432 records were initially retrieved through comprehensive searches of databases including PubMed, MEDLINE, Embase, Scopus, CENTRAL, and Science Citation Index. After removing 127 duplicate entries, 305 records were carried forward for screening. During the title and abstract review, 243 studies were excluded - 158 were unrelated to regenerative endodontics, 55 did not involve laser-based interventions, and 30 lacked assessments of relevant GF outcomes.The remaining 62 full-text articles were then evaluated in detail for eligibility. Of these, 47 were excluded for various reasons: nine lacked full-text availability; six were published in languages other than English; 14 were reviews, letters to the editor, or conference abstracts; and 18 were animal studies. Following this rigorous selection process, 15 studies fulfilled all inclusion criteria and were incorporated into the final qualitative synthesis. The entire selection process is summarized in the PRISMA flow diagram (Figure 1).

Study Characteristics

Publication Years and Study Designs: The studies included in this review were published between 2014 and 2025. Thirteen of the fifteen studies were in vitro investigations. One study combined in vitro experiments with an in vivo rodent model, while another utilized an ex vivo 3D human dentin-pulp complex culture. These preclinical designs provided controlled environments to evaluate the effects of laser therapy on cellular responses relevant to regenerative endodontics.

Tissue and Cell Sources:* *All included studies used human-derived biological samples. Dental pulp stem cells (DPSCs) were the most employed cell type, followed by stem cells from the apical papilla (SCAPs), human dentin discs, and pulpal fibroblasts. One study utilized human dentin-pulp complex tissue in a 3D culture system to mimic clinical tissue architecture.

Laser Types and Parameters: Diode lasers were the most frequently studied, with wavelengths ranging from 450 nm to 980 nm. Out of the diode laser wavelengths tested, the 660 nm showed the most consistent and measurable advantages in angiogenic stimulation, such as a 15.3-fold increase in VEGF and a 6.1-fold increase in dentin sialophosphoprotein (DSPP), at energy densities of 1 to 3.7 J/cm². The 810 nm showed the most promising results in dentinogenic activity, with a 17.7-fold increase in DMP1, at energy densities of 1 to 3 J/cm², five minutes. These wavelengths are within the range of the red/near-infrared spectrum, which aligns with the photobiomodulation theory. Two studies investigated the effects of Er:YAG lasers at 2,940 nm. Energy densities ranged widely, from as low as 0.39 J/cm² to over 10 J/cm². Exposure durations, power outputs, and application frequencies varied significantly across studies. Some employed single-dose irradiation protocols, while others tested multiple exposures or dual wavelengths. Such variation underscores the current lack of standardization in laser-based regenerative protocols.

Measured Outcomes

The primary focus across studies was the expression of GFs associated with angiogenesis, stem cell activity, and tissue remodeling. Commonly measured biomarkers included VEGF, TGF-β1, PDGF, bFGF, and BMPs. In addition, odontogenic and osteogenic markers such as DSPP, dentin matrix protein 1 (DMP1), alkaline phosphatase (ALP), and runt-related transcription factor 2 (RUNX2) were frequently assessed. A few studies also evaluated markers of neural differentiation or stemness to explore broader regenerative potential.

Table 1 presents a comprehensive summary of the studies included in this review.

**Table 1:** Summary of included studies ALP: Alkaline phosphatase; BMP: Bone morphogenetic protein; BSP: Bone sialoprotein; DSPP: Dentin sialophosphoprotein; DSP: Dentin sialoprotein; DPSC: Dental pulp stem cell; DMP: Dentin matrix protein; EDTA: Ethylenediaminetetraacetic acid; FGF: Fibroblast growth factor; GaAlAs: Gallium-aluminum-arsenide; GF: Growth factor; IGF: Insulin-like growth factor; LLLT: Low-level laser therapy; MMP: Matrix metalloproteinase; PDGF: Platelet-derived growth factor; RUNX2: Runt-related transcription factor 2; RANKL: Receptor activator of nuclear factor kappa-B ligand; SOX2: SRY-box transcription factor 2; TNF: Tumor necrosis factor; TGF: Transforming growth factor; VLT: Visible light therapy; VEGF: Vascular endothelial growth factor

No.	Author (Year)	Design	Cell/Tissue	Laser (Wavelength)	Energy	GFs	Key outcomes	Regenerative indicators	Conclusion
1	Arany et al. (2014) [8]	In vitro and in vivo	Human DPSCs	Diode (GaAlAs laser diode system 810 nm)	3 J/cm², 5 minutes	TGF-β1 (latent)	Activated latent TGF-β1, resulting in increased cell migration and differentiation	Angiogenesis, dentin bridge formation	Demonstrated potential of diode laser to activate latent TGF-β1, enhancing pulp-dentin regeneration
2	Tabatabaei et al. (2015) [12]	In vitro	DPSCs	Diode (810 nm)	0.5 and 1.0 J/cm²	ALP, osteogenic markers	Increased cell proliferation and osteogenic differentiation	Osteogenic differentiation	Suggested low-level diode laser promotes osteogenic differentiation of DPSCs
3	El Nawam et al. (2019) [9]	Ex vivo 3D culture	Human dentin–pulp complex	Diode (660 and 810 nm)	1 and 3 J/cm²	VEGF, VEGFR2, DSPP, DMP1, BSP	660 nm laser increased VEGF by 15.3 times and DSPP by 6.1 times; 810 nm increased DMP1 by 17.7 times	Angiogenesis, dentinogenesis	Confirmed wavelength-dependent modulation of angiogenic and dentinogenic markers
4	Vitor et al. (2020) [10]	In vitro	Human pulpal fibroblasts	Diode (660 nm)	2.5 and 3.7 J/cm²	VEGF-A, VEGF-C, VEGFR1, BMP-9, PDGF, FGF-2	Laser stimulation led to increased VEGF-A, VEGF-C, VEGFR1, and BMP-9 expression; formation of capillary-like structures	Angiogenesis, vascular response	Showed diode laser enhances angiogenesis and vascular signaling
5	Akhila et al. (2021) [13]	In vitro	Human dentin discs	Diode (660 nm)	6 J (100 mW, 60 seconds)	TGF-β1	LLLT released more TGF-β1 compared to EDTA or saline	Bioactive matrix activation	Indicated diode laser superior in releasing TGF-β1 compared to conventional irrigants
6	Rahmati et al. (2022) [14]	In vitro	SCAPs	Diode (epic10, BIOLASE, 940nm)	4 J/cm²	DSPP, DMP-1, ALP, BSP	Upregulation of osteogenic and odontogenic markers	Enhanced stem cell differentiation	Supported laser-mediated stimulation of odontogenic/osteogenic differentiation in SCAPs
7	da Rocha et al. (2022) [15]	In vitro	DPSCs	Photobiomodulation (808 nm)	Not specified	RUNX2, ALP, TNF, RANKL, MMPs	Suppressed inflammatory genes; increased mineralization-associated genes	Odontogenic potential under inflammatory challenge	Suggested PBM modulates inflammation and promotes mineralization genes
8	Martín et al. (2023) [16]	In vitro	DPSCs	Diode (808 and 980 nm)	1.5 W, 45 J/cm²	DSPP, DMP1, TGF-β1	Laser with EDTA resulted in decreased DSPP and DMP1 expression; TGF-β1 unchanged	Dentinogenesis modulation	Highlighted possible inhibitory effect of diode laser + EDTA on dentinogenic markers
9	Yarita et al. (2024) [17]	In vitro	hDPSCs	Semiconductor (650 nm)	10, 30, 150 mW; 40 sec	ALP, DSPP, DMP-1, NESTIN	Increased proliferation and odontoblastic differentiation	Odontoblast-like cell differentiation	Confirmed diode laser enhances odontoblastic differentiation
10	Hancerliogullari, Erdemir, Kisa (2024) [18]	In vitro	Human dentin	Er:YAG (2940 nm)	30 mJ, 2 W, 20 Hz, 10 sec	TGF-β1, IGF-1, BMP-7, VEGF-A	Laser with EDTA led to greater TGF-β1 and BMP-7 release than EDTA alone	GF release from dentin	Demonstrated Er:YAG + EDTA synergistically enhances GF release
11	García-Guerrero et al. (2024) [19]	In vitro	Human dentin	Diode (650 and 810 nm)	100 mW, 60 seconds	TGF-β1, VEGF, PDGF-BB	Laser conditioning enhanced release of bioactive molecules from dentin	GF-mediated matrix activation	Suggested diode laser improves dentin bioactivity by enhancing GF release.
12	Malekpour et al. (2024) [6]	In vitro (2D and 3D)	DPSCs	Diode (660 nm)	2.5 and 3.7 J/cm²	VEGF, βIII-tubulin	Increased VEGF expression and neural morphology in 3D culture	Angiogenesis, early neurogenesis	Indicated potential for laser to stimulate angiogenesis and neurogenic differentiation.
13	Yoshida et al. (2025) [20]	In vitro	DPSCs	Er:YAG (2940 nm)	0.39 J/cm², 50 mJ	TGF-β1, SMAD3, RUNX2, DSPP, ALP	Laser with VLT enhanced SMAD3 expression, ALP activity, and mineralization	Pulp-dentin complex regeneration	Provided evidence for Er:YAG laser in supporting mineralization and pulp–dentin regeneration.
14	Alborzy et al. (2025) [21]	In vitro	DPSCs	Diode (450 and 980 nm)	2 and 4 J/cm²	OCT4, SOX2, NANOG, NESTIN	450 nm laser with Biodentine significantly increased proliferation and pluripotency	Stemness and regenerative enhancement	Suggested diode laser + Biodentine synergistically enhances stemness.
15	Rezaei et al. (2025) [7]	In vitro	DPSCs	Diode (660 nm)	3 J/cm²	VEGF, DSP	Double dose resulted in increased cell proliferation and VEGF and DSP expression	Angiogenesis, dentinogenesis	Supported dose-dependent stimulation of angiogenic and dentinogenic markers.

Risk of Bias Assessment

The methodological quality of the included studies was evaluated using a 12-point QUIN checklist designed for in vitro studies [13]. Overall, the studies demonstrated low risk of bias across the accessed domains. All studies clearly stated their research aims and provided appropriate justification for the in vitro models used. Sample characterization, group allocation, and the use of control groups were consistently reported. Outcome measures were relevant and clearly defined, and statistical analyses were typically appropriate for the study design.

However, two key limitations emerged across the dataset. First, none of the studies reported conducting sample size calculations. Second, blinding of outcome assessment was either not implemented or not mentioned.

Table 2 summarizes the risk of bias scores of the included studies.

**Table 2:** Risk of bias of included studies

Criterion	1. Clearly stated aim/objective	2. In vitro model justification	3. Sample size calculation	4. Sample characterization	5. Group allocation	6. Control group used	7. Blinding	8. Outcome measure appropriateness	9. Statistical analysis	10. Reproducibility of method	11. Results presented appropriately	12. Conflict of interest and funding disclosed	Total Score (out of 24)	Overall Risk of Bias
Arany et al. (2014) [8]	2	2	0	2	2	2	0	2	2	2	2	2	20	Low
Tabatabaei et al. (2015) [14]	2	2	0	2	2	2	0	2	2	2	2	2	20	Low
El Nawam et al. (2019) [9]	2	2	0	2	2	2	0	2	2	2	2	2	20	Low
Vitor et al. (2020) [10]	2	2	0	2	2	2	0	2	2	2	2	2	20	Low
Akhila et al. (2021) [15]	2	2	0	2	2	2	1	2	2	2	2	2	21	Low
Rahmati et al. (2022) [16]	2	2	0	2	2	2	0	2	2	2	2	2	20	Low
da Rocha et al. (2022) [17]	2	2	0	2	2	2	0	2	2	2	2	2	20	Low
Martín et al. (2023) [18]	2	2	0	2	2	2	0	2	2	2	2	2	20	Low
Yarita et al. (2024) [19]	2	2	0	2	2	2	0	2	2	2	2	2	20	Low
Hancerliogullari, Erdemir, Kisa (2024) [20]	2	2	0	2	2	2	0	2	2	2	2	2	20	Low
García-Guerrero et al. (2024) [21]	2	2	0	2	2	2	0	2	2	2	2	2	20	Low
Malekpour et al. (2024) [6]	2	2	0	2	2	2	0	2	2	2	2	2	20	Low
Yoshida et al. (2025) [22]	2	2	0	2	2	2	0	2	2	2	2	2	20	Low
Alborzy et al. (2025) [23]	2	2	0	2	2	2	0	2	2	2	2	2	20	Low
Rezaei et al. (2025) [7]	2	2	0	2	2	2	0	2	2	2	2	2	20	Low

Discussion

This systematic review explored the effects of laser therapy on REPs, particularly focusing on GF modulation, stem cell behaviors, scaffold interaction, and angiogenesis/neurogenesis. The findings across various studies show a consistent biological impact of laser therapy, particularly in the activation of GFs and the promotion of stem cell differentiation, which align with the broader body of literature on laser-assisted tissue regeneration.

Activation and Release of GFs

Multiple studies have demonstrated that laser therapy, particularly LLLT, significantly enhances the release of GFs such as VEGF and TGF-β1, which are critical for tissue regeneration. For example, El Nawam et al., Vitor et al., Malekpour et al., and Rezaei et al. observed that diode laser application upregulated VEGF expression, promoting angiogenesis [6,7,9,10]. Additionally, Arany et al. and Akhila et al. reported the activation of TGF-β1, which is known to aid in collagen synthesis and extracellular matrix remodeling [8,15]. These findings are in line with previous research suggesting that laser therapy can enhance cellular signaling and promote regenerative responses in a variety of tissues, particularly in the context of dental pulp regeneration.

Stem Cell Proliferation and Differentiation

Laser therapy’s impact on stem cell proliferation and differentiation is another important finding from the reviewed studies. The upregulation of odontogenic markers such as DSPP and dentin matrix protein 1 (DMP1) observed in studies by El Nawam et al., Rahmati et al., and Yarita et al. suggests that laser application supports the differentiation of stem cells into odontoblast-like cells, contributing to dentin formation [9,16,19]. Similarly, studies by Tabatabaei et al. and Rahmati et al. demonstrated increased osteogenic markers (ALP, RUNX2, bone sialoprotein (BSP)), indicating that laser therapy could promote osteogenesis, supporting hard tissue regeneration in REPs [14,16]. This evidence supports the notion that laser therapy not only promotes tissue regeneration but also actively directs stem cells towards differentiation into tissue-specific lineages.

Action on Scaffold

Laser therapy also affects the interaction between scaffolds and the regenerative environment. In studies by García-Guerrero et al. and Hancerliogullari et al., the combination of laser therapy with Biodentine enhanced the release of GFs from dentin, providing a more favorable environment for tissue regeneration [20,21]. Similarly, Alborzy et al. demonstrated that diode lasers, in conjunction with Biodentine, boosted the expression of stemness markers, further supporting the role of lasers in optimizing scaffold function for tissue regeneration [23]. This suggests that laser therapy can enhance the bioactivity of scaffolds, making them more effective in promoting cell attachment and differentiation.

Angiogenesis and Neurogenesis

Several studies explored the effects of laser therapy on angiogenesis and neurogenesis, which are crucial for the successful regeneration of the pulp-dentin complex. Malekpour et al. observed increased expression of βIII-tubulin, a marker for neurogenic differentiation, following diode laser treatment [6]. In addition, Alborzy et al. demonstrated that laser exposure increased pluripotency markers like OCT4, SOX2, and NESTIN, indicating the potential for both neurogenic and angiogenic stimulation [23]. These findings support the idea that laser therapy may facilitate both vascular and neural integration, which are essential for the functional restoration of the pulp.

Laser Modalities and Mechanisms of Action

Across the included studies, diode lasers emerged as the most widely employed modality. Their wavelengths ranged from 450 to 980 nm, and the majority operated within the red to near-infrared spectrum, aligning with the principles of LLLT. LLLT refers to the therapeutic application of low-power laser energy to induce photobiomodulation, a process through which light is absorbed by cellular chromophores, particularly mitochondrial cytochrome c oxidase, thereby triggering intracellular signaling cascades, increased adenosine triphosphate (ATP) production, and subsequent gene transcription [24]. Although diode lasers were commonly used for LLLT in these studies, several applications employed parameters that diverged from the conventional criteria typically associated with LLLT. Additionally, two studies employed Er:YAG lasers, which operate at higher wavelengths (2,940 nm) and interact with tissues primarily through water absorption, suggesting distinct biological effects [25].

Limitations

The evidence in this review has several limitations. Most studies were conducted in vitro, under controlled conditions that do not replicate the complexity of the human clinical environment. For instance, El Nawam et al. and Vitor et al. used cell cultures, which lack the dynamic interactions seen in live tissues [9,10]. Additionally, there was limited in vivo data, with only one study (Arany et al.) providing in vivo insights, and another using an ex vivo model (El Nawam et al.) [8,9]. Finally, the variation in laser parameters across studies, such as wavelength, power, and exposure time, makes it difficult to determine the optimal laser settings for clinical use.

The review process also had limitations. First, studies not published in English were excluded, which could introduce language bias and omit relevant research. Many studies also lacked sample size justification or transparency in their methodology, such as blinding or randomization, which may affect the reliability and generalizability of the findings, though the risk of bias was considered low.

Implications for practice, policy, and future research

The findings from this review highlight the potential of laser therapy as a promising adjunct in regenerative endodontics. Clinicians should consider incorporating laser therapy into regenerative procedures, particularly given its ability to modulate GF activity and promote stem cell differentiation. However, the clinical implications remain uncertain without further well-controlled human trials to confirm these preclinical findings.

From a policy perspective, the integration of laser therapy into clinical practice would require further validation, including cost-effectiveness studies to assess its value in routine practice. Standardizing laser protocols and conducting long-term clinical trials are crucial for broader clinical adoption.

Future research should prioritize well-designed human trials, focusing on evaluating long-term outcomes such as root maturation, pulp vitality restoration, and apical healing. Furthermore, research should aim to standardize laser parameters and explore the synergistic effects of lasers with various bioactive scaffolds.

## Conclusions

This systematic review explored the potential of laser therapy to improve regenerative endodontic outcomes through GF modulation. Preclinical evidence consistently demonstrates that lasers can enhance the expression of key signaling molecules involved in tissue regeneration and healing. However, the clinical implications remain uncertain due to the lack of well-controlled human trials. The review acknowledges limitations such as the predominance of animal studies and the need for more robust clinical evidence. Future research should focus on well-designed human trials to confirm these findings, optimize treatment protocols, and evaluate the long-term impact of laser therapy on regenerative endodontics.
